# Embryonic Cardiomyocyte, but Not Autologous Stem Cell Transplantation, Restricts Infarct Expansion, Enhances Ventricular Function, and Improves Long-Term Survival

**DOI:** 10.1371/journal.pone.0061510

**Published:** 2013-04-09

**Authors:** Leonie E. Paulis, Alexandra M. Klein, Alexander Ghanem, Tessa Geelen, Bram F. Coolen, Martin Breitbach, Katrin Zimmermann, Klaas Nicolay, Bernd K. Fleischmann, Wilhelm Roell, Gustav J. Strijkers

**Affiliations:** 1 Biomedical NMR, Department of Biomedical Engineering, Eindhoven University of Technology, Eindhoven, The Netherlands; 2 Institute of Physiology I, Life and Brain Centre, University of Bonn, Bonn, Germany; 3 Department of Medicine/Cardiology, University of Bonn, Bonn, Germany; 4 Department of Pharmacology and Toxicology, Biomedical Center, University of Bonn, Bonn, Germany; 5 Department of Cardiac Surgery, University of Bonn, Bonn, Germany; Institute of Clinical Medicine, National Cheng Kung University, Taiwan

## Abstract

**Aims:**

Controversy exists in regard to the beneficial effects of transplanting cardiac or somatic progenitor cells upon myocardial injury. We have therefore investigated the functional short- and long-term consequences after intramyocardial transplantation of these cell types in a murine lesion model.

**Methods and Results:**

Myocardial infarction (MI) was induced in mice (n = 75), followed by the intramyocardial injection of 1−2×10^5^ luciferase- and GFP-expressing embryonic cardiomyocytes (eCMs), skeletal myoblasts (SMs), mesenchymal stem cells (MSCs) or medium into the infarct. Non-treated healthy mice (n = 6) served as controls. Bioluminescence and fluorescence imaging confirmed the engraftment and survival of the cells up to seven weeks postoperatively. After two weeks MRI was performed, which showed that infarct volume was significantly decreased by eCMs only (14.8±2.2% MI+eCM vs. 26.7±1.6% MI). Left ventricular dilation was significantly decreased by transplantation of any cell type, but most efficiently by eCMs. Moreover, eCM treatment increased the ejection fraction and cardiac output significantly to 33.4±2.2% and 22.3±1.2 ml/min. In addition, this cell type exclusively and significantly increased the end-systolic wall thickness in the infarct center and borders and raised the wall thickening in the infarct borders. Repetitive echocardiography examinations at later time points confirmed that these beneficial effects were accompanied by better survival rates.

**Conclusion:**

Cellular cardiomyoplasty employing contractile and electrically coupling embryonic cardiomyocytes (eCMs) into ischemic myocardium provoked significantly smaller infarcts with less adverse remodeling and improved cardiac function and long-term survival compared to transplantation of somatic cells (SMs and MSCs), thereby proving that a cardiomyocyte phenotype is important to restore myocardial function.

## Introduction

Myocardial infarction is characterized by extensive necrosis of cardiomyocytes, which causes a non-reversible loss of rhythmic contractile abilities [1]. The low proliferative capacity of terminally differentiated cardiomyocytes has led to the exploration of stem cell-based therapies to regenerate myocardium and reduce the occurrence of heart failure [2].

Intramyocardial injection of embryonic cardiomyocytes (eCMs) in mice has previously been shown to result in electrical coupling to native myocardium, improved ventricular function and reduction of ventricular arrhythmias [3]–[6]. The combination of these key functional characteristics of eCMs is unique compared to already clinically applied somatic cells such as skeletal myoblasts (SMs) and mesenchymal stem cells (MSCs). For these cell types neither electromechanical integration into the heart nor transdifferentiation into cardiomyocytes has been unequivocally proven [7], [8]. Therefore, beneficial effects seen in some animal studies and human trials after cardiac SM or MSC transplantation [9], [10] have been preferentially attributed to passive mechanisms such as the preservation of cardiac compliance or paracrine effects [11].

Nevertheless, this area is still controversial and the best-suited cell type has not been identified so far. Therefore, the goal of this study was to investigate in detail global and regional cardiac contractile function following cellular cardiomyoplasty. In particular we aimed to address, if observed changes were dependent on the ability of the cells to contract and couple electrically to native myocardium. Therefore, contractile and electrically coupling eCMs were compared to, on the one hand, contractile but not electrically coupling SMs and, on the other hand, neither contractile nor electrically coupling MSCs. The cells were transplanted into infarcted syngeneic hearts of two different mouse strains (CD1 and C57BL/6).

To evaluate myocardial function, we applied a unique set of imaging and analysis tools to investigate local and global cardiac contractile function and geometry, cell survival, and long-term outcome. High spatial- and temporal-resolution in vivo magnetic resonance imaging (MRI) was performed to characterize global cardiac function [12]. Moreover, to investigate active enhancement of local contractility by the transplanted cells throughout the cardiac cycle, data were evaluated by use of the AHA 16-segment model, where for each segment 45–50 radial chords were analyzed. Furthermore, using late gadolinium enhancement (LGE) MRI, infarcted and remote myocardium could be discriminated clearly [13]. Additionally, survival of transplanted cells, cardiac morphology and function were monitored up to 7 weeks by bioluminescence imaging (BLI) and three-dimensional echocardiography.

## Methods

All animal experiments were performed in accordance with the declaration of Helsinki and were approved by the local ethical committees for animal experiments of Nordrhein-Westfalen (LANUV) and the University of Maastricht.

### Cell isolation and culture

eCMs and SMs were obtained from transgenic CD-1 and C57BL/6 mouse embryos, expressing green fluorescent protein (GFP) under the α-actin promoter as described [4], [14], [15]. eCMs were isolated from embryonic hearts at E13.5, whereas for SM isolation the diaphragm was used at E18.5. Transgenic MSCs were aspirated from the femur and tibia of adult CD-1 and C57BL/6 mice, with GFP expression under control of the α-actin promoter [7].

### Viral transduction of cells

Cells underwent lentiviral transduction prior to intracardiac injection to introduce viral-enhanced firefly luciferase (veff-luc) expression. Veff-luc was cloned into a pRRL-SIN18 backbone and its expression was driven by a cytomegalovirus promoter. Four hours after isolation, cells were incubated at approximately 1.5×10^6^ viral particles/2.5×10^5^ cells for 10–12 h at 37°C. Subsequently, cells were washed with phosphate buffered saline (PBS, Sigma-Aldrich), harvested with trypsin (Gibco) and resuspended in IMDM-medium at a concentration of 1−2×10^5^ cells/5 µL.

### Myocardial infarction and intramyocardial cell transplantation

Transmural myocardial infarctions were generated in wild type male CD-1 (n = 28) and C57BL/6 mice (n = 47) (10–12 weeks old, Charles River Laboratories, Sulzfeld, Germany). Non-treated healthy CD-1 mice (n = 6) served as controls. Under general anesthesia, mice were intubated and mechanically ventilated as described before [4], [14]. The heart was exposed by a left lateral thoracotomy and the left coronary artery was permanently occluded directly distal to the left atrial auricle. Thereafter, 1−2×10^5^ cells or 5 µL IMDM-medium were injected into the border zones and center of the ischemic area. To visually confirm intramyocardial delivery, a blue dye was co-injected.

### Experimental setup

Two different protocols were used to evaluate the effects of cell transplantation on cardiac remodeling after myocardial infarction. 1) Short-term effects were studied by MRI 2 weeks after myocardial infarction in CD-1 mice treated with eCMs (MI+eCM), SMs (MI+SM), MSCs (MI+MSC) or IMDM-medium (MI). As a reference, age and gender matched CD-1 mice were used (no MI). 2) To monitor long-term cell survival and cardiac remodeling, BLI and/or echocardiography were performed at day 5, 13 and 49 after coronary artery ligation and transplantation of eCMs, SMs or MSCs (isolated from C57BL/6 mice). For long-term experiments inbred C57BL/6 mice were used to avoid rejection of transplanted cells.

### *In vivo* MRI

Cardiac MRI was performed with a 9.4 T scanner (Bruker Biospin GmbH, Ettlingen, Germany). Mice were anesthetized with isoflurane and placed in supine position in a 35-mm-diameter birdcage RF-coil (Rapid Biomedical, Rimpar, Germany). Electrocardiogram (ECG), respiration and temperature (Small Animal Instruments Inc., Stony Brook, USA) were continuously monitored.

Infarct size was assessed by LGE MRI 5 min after intravenous administration of 0.3 mmol Gd-DTPA/kg bodyweight (Bayer HealthCare Pharmaceuticals, Mijdrecht, the Netherlands) with an ECG-triggered and respiratory-gated T_1_-weighted multi-slice FLASH sequence in short-axis orientation (TR = 63 ms, TE = 1.8 ms, FA = 60°, FOV = 3×3 cm^2^, matrix = 192×192, NEX = 6, slice thickness = 1 mm, number of slices = 9). For evaluation of cardiac morphology and function, cine MR-images in short- and long-axis orientations were acquired with a single-slice FLASH sequence (TR = 7 ms, TE = 1.8 ms, FA = 15°, FOV = 3×3 cm^2^, matrix = 192×192, NEX = 6, slice thickness = 1 mm). Ten to 11 slices were acquired in short-axis orientation to cover the heart from apex to base.

### MRI data analysis

Epi- and endocardial contours of the left ventricle were traced semi-automatically [16] in short-axis LGE images and short- and long-axis cine images in end-diastole (ED) and end-systole (ES) using CAAS MRV FARM 2.0 software (Pie Medical Imaging, Maastricht, the Netherlands). To determine the infarct size, hyperintense voxels within the segmented left ventricle on T_1_-weighted LGE MR-images were identified with a threshold algorithm. The threshold value was defined as the mean signal intensity within a septal region of interest plus five times its standard deviation. The transmural infarct volume was calculated as the volume of infarcted myocardium relative to the total left ventricular (LV) volume. For quantification of the epicardial infarct area, the left ventricle was partitioned into 100 segments per slice. Segments in which infarct transmurality exceeded 50% were assigned as infarct. The epicardial circumferential length of the infarcted segments was calculated with home-built software in Mathematica 6.0 (Wolfram Research Europe, Oxfordshire, United Kingdom) and was multiplied with the slice thickness to obtain the infarct surface area. The epicardial infarct surface area was calculated as the area of infarcted myocardium relative to the total LV area.

LV dimensions were described by the endocardial ED and ES volume (EDV and ESV, respectively) on cine images, which were also used to calculate global functional parameters as the ejection fraction (EF) and cardiac output (CO). Regional contractility was evaluated by applying a three-dimensional centerline model to the segmented LV [17]. In each short-axis slice 100 radial chords were calculated that represented the wall thickness (WTS) perpendicular to the myocardial wall. Wall thickening was defined as WT = (WTS_ES_−WTS_ED_)/WTS_ES_. Regional WTS and WT were evaluated by distributing all chords according to the standard American Heart Association (AHA) model [18].

### In vivo BLI

Bioluminescence images of luciferase expression were acquired under isoflurane anesthesia 10 min after intraperitoneal injection of D-luciferin (25 mg/mouse, AppliChem, Darmstadt, Germany) with a Xenogen IVIS200 system (Caliper Life Sciences, Hopkinton, USA). Bioluminescence signals were detected for 90 s with a charge-coupled device camera. Mice transplanted with non-transduced cells served as control.

### In vivo echocardiography

Echocardiography was performed on an HDI-5000 (ATL Philips Medical Systems, Best, the Netherlands). Mice were anesthetized with a low-dose of isoflurane and 2D M-mode images were acquired in short-axis position at 0.5 mm intervals from apex to base and in long-axis position using a 15 MHz linear-array transducer allowing frame rates up to 230 Hz. To calculate functional cardiac parameters, the endocardium was segmented in ED and ES in both the long-axis image and a single short-axis image at the level of the papillary muscle.

### Ex vivo histology

Mice were sacrificed by cervical dislocation. Hearts were perfused with PBS, followed by fixation with 4% paraformaldehyde (Sigma-Aldrich) and dehydration in 20% sucrose (Sigma-Aldrich). Short-axis cryosections (10 µm) were prepared at 50 µm intervals with a CM3050s cryostat (Leica GmbH, Wetzlar, Germany).

Cell engraftment and differentiation were evaluated ex vivo using an Axiovert 40CFL fluorescence microscope combined with an AxioCam MRm camera (Carl Zeiss MicroImaging GmbH, Göttingen, Germany). To identify engrafted cells and to exclude autofluorescence, GFP expression was detected with a double filter (FITC/Cy3), using optimized exposure times.

To assess differentiation, cryosections were fluorescently labeled with rabbit-anti-mouse connexin43 (1∶1000, Peptide Specialty Laboratories GmbH, Heidelberg, Germany) coupled to donkey-anti-rabbit DyLight549 (1∶400, Jackson ImmunoResearch, Suffolk, United Kingdom), mouse-anti-human α-actinin (1∶400, Sigma-Aldrich) conjugated to goat-anti-mouse Cy5 (1∶400, Jackson ImmunoResearch) and rat-anti-mouse CD73 (1∶400, BD Pharmingen. Heidelberg, Germany) combined with goat-anti-rat Alexa555 (Invitrogen, Darmstadt, Germany). For nuclear staining Hoechst 33342 (1∶1000, Sigma-Aldrich) was used.

Collagen was stained with direct red 80 in picric acid (1 g/L, both Sigma-Aldrich). Images were acquired with a ProgRes C10 in combination with Capture Pro Image Acquisition Software (JenOptik Optical Systems GmbH, Jena, Germany) through a binocular loupe (Leica) with 10 ms shutter time.

### Statistical analysis

Data were presented as mean±SEM. One-way analysis of variance (ANOVA) with Dunnett's correction for intergroup comparisons was used to compare MRI data of healthy mice and eCM-, SM- or MSC-treated mice to untreated mice. One-way ANOVA with Dunnett's correction was used to detect differences between eCM- and SM- or MSC-treated mice. Echocardiography data were analyzed with repeated measures ANOVA to detect changes in eCM, SM or MSC transplanted mice. For statistical analysis of the time-course of survival, a log-rank test was used. Level of statistical significance was set at α  = 0.05.

## Results

### Cell survival and differentiation

**Figure 1:**
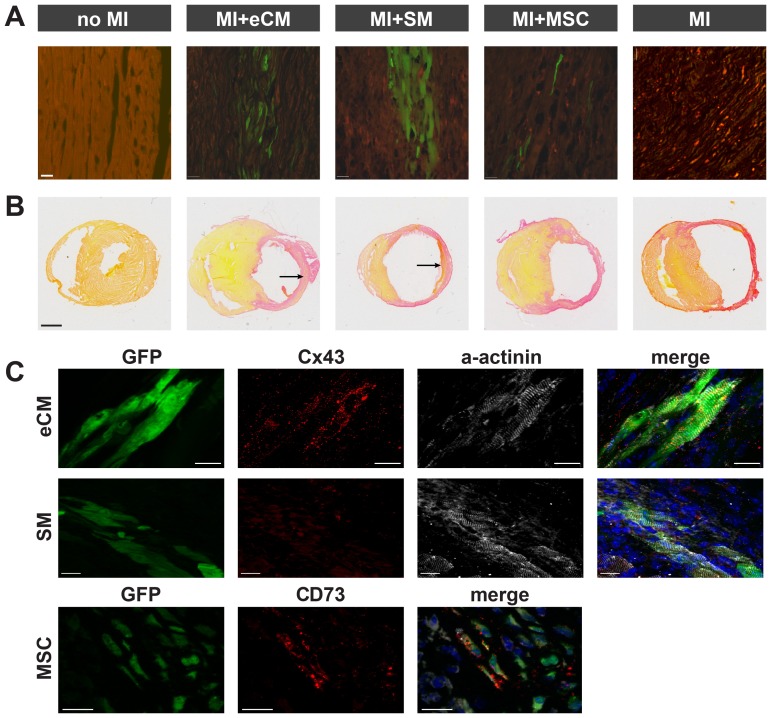
Fluorescence microscopy and histology of myocardial tissues of treatment and control groups. A) Ex vivo fluorescence microscopy of GFP expressing eCMs, SMs and MSCs two weeks after transplantation in infarcted myocardium to evaluate cell engraftment and survival. GFP is depicted in green and autofluorescence is shown in red. Scale bar = 20 µm. B) Ex vivo picrosirius red staining to determine general infarct composition two weeks postoperatively. Engrafted eCMs and SMs can be identified within the lesion (arrows). Scale bar = 1 mm. C) Ex vivo fluorescence microscopy to evaluate cell differentiation at 7 weeks (no MI, MI+eCM, MI+SM, MI) or 2 weeks (MI+MSC) after surgery and transplantation. For all groups, GFP fluorescence is depicted in green and cell nuclei in blue. For eCM (top) and SM (middle) the cardiomyocyte marker Connexin43 is shown in red and α-actinin in white, whereas for MSC (bottom) the mesenchymal stem cell marker CD73 is shown in red. Scale bars = 20 µm.

Ischemic, necrotic myocardium is a challenging environment for the engraftment and survival of transplanted cells. Two and 7 weeks after implantation, the presence of grafted cells within the lesion was proven by their native GFP fluorescence (Figs. 1A and C). Bright epifluorescence of GFP-expression from eCMs (MI+eCM), SMs (MI+SM) or MSCs (MI+MSC) was detected in the infarct. No GFP-fluorescence was observed in infarcted hearts of mice that did not receive cell injections (MI) and in the anterolateral LV wall of mice without MI (no MI). The grafted cells were found exclusively within the infarcted myocardium and in the border zone of the lesion and did not migrate into remote myocardium.

The size of the grafts was dependent on the type of cells transplanted. Similar as reported earlier by our group overall cell survival was rather poor [4]. To estimate number of engrafted GFP+ cells we counted the cells and this yielded only a few thousand in the SM (n = 3) and in the eCM grafts (n = 3) [4]. For MSC only singular engrafted cells were detected (Fig. 1A). One mouse in which cell engraftment had failed was excluded from further analysis. Mice with successful engraftment of eCMs (n = 5), SMs (n = 7) and MSCs (n = 7) were included.

In Fig. 1B, picrosirius histological staining illustrates the effect of cell transplantation on the architecture of the left ventricle 2 weeks postoperatively. Compared to wild-type mice (no MI), LV dilation and wall thinning at the lesion site were most prominent in mice with myocardial infarction that were not treated (MI). After intramyocardial injection of SMs (MI+SM) or MSCs (MI+MSC) only a slight reduction of LV dilation was observed. After transplantation of eCMs (MI+eCM) dilation of the left ventricle was clearly more attenuated compared to the other groups. Furthermore, LV wall thickness within the infarct was increased by the engraftment of eCMs, which like the SMs could be identified within the lesion by picrosirius red staining as islets of viable cells.

In transplanted eCMs the gap junction protein Connexin43 as well as α-actinin expression could be demonstrated 2 and 7 weeks postoperatively. Data at 7 weeks are presented in Fig. 1C. Engrafted cardiomyocytes showed prominent cross-striation indicating further differentiation of the transplanted eCMs [4]. As expected, grafted SMs only stained positive for α-actinin, but not for Connexin43, indicating their differentiation into mature myocytes. MSCs did not adopt a contractile phenotype and, moreover, still expressed the MSC-marker CD73 [7].

### Infarct volume and area

**Figure 2:**
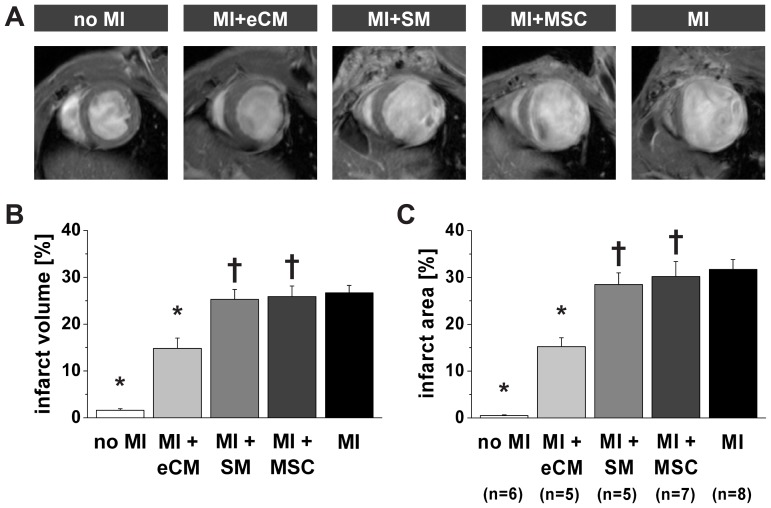
Quantification of infarct size by MRI. A) Representative in vivo T_1_-weighted mid-LV short-axis LGE MR-images two weeks after myocardial infarction for each experimental group. Infarct size was quantified from LGE images as B) relative transmural infarct volume and C) relative epicardial infarct surface area. No MI: n = 6, MI+eCM: n = 5, MI+SM: n = 5, MI+MSC: n = 7 and MI: n = 8. *  =  p<0.05 vs. MI, †  =  p<0.05 vs. MI+eCM. The infarct volume and area measured in wild type mice (no MI) was indicative for the accuracy of the analysis.

Histology established differences in cell engraftment and survival, as well as changes in infarct remodeling. In order to quantify the impact on cardiac function in vivo, high-resolution MRI scans were performed 2 weeks post-operatively. Representative LGE short-axis MR-images for the various groups are shown in Fig. 2A. These images were obtained at the mid-LV level, making them therefore very comparable. The hyperenhanced infarcted myocardium could clearly be delineated in mice with MI, MI+SM and MI+MSC, whereas in mice with MI+eCM an inhomogeneous pattern of epicardial signal enhancement was observed in the anterolateral wall.

Quantification of the relative transmural infarct volume (Fig. 2B) indicated that transplantation of eCMs after myocardial infarction led to a significant decline in the infarct volume from 26.7±1.6% (MI, n = 8) to 14.8±2.2% (MI+eCM, n = 5). This was a unique feature of eCM therapy, since intramyocardial injection of SMs (n = 5) or MSCs (n = 7) had no significant effect on the infarct volume (25.3±2.1% and 25.9±2.3%, respectively, p>0.05 vs. MI). Another common measure of chronic infarct size is the epicardial surface area of LV segments consisting of more than 50% infarcted myocardium (Fig. 2C). The relative infarct area showed a similar trend as the infarct volume (compare Figs. 2B and C). The relative epicardial infarct area was significantly reduced by eCM treatment from 31.7±2.1% (MI) to 15.2±1.9% (MI+eCM, p<0.05), but no significant changes were found in case of SM or MSC injection (28.5±2.5% and 30.2±3.2%, respectively). Furthermore, when comparing the effect of transplantation of eCMs to that of SMs and MSCs, the infarct volume and area were both significantly lowered by eCM engraftment (p<0.05, MI+eCM vs. MI+SM and MI+MSC).

### Global cardiac morphology and function

**Figure 3:**
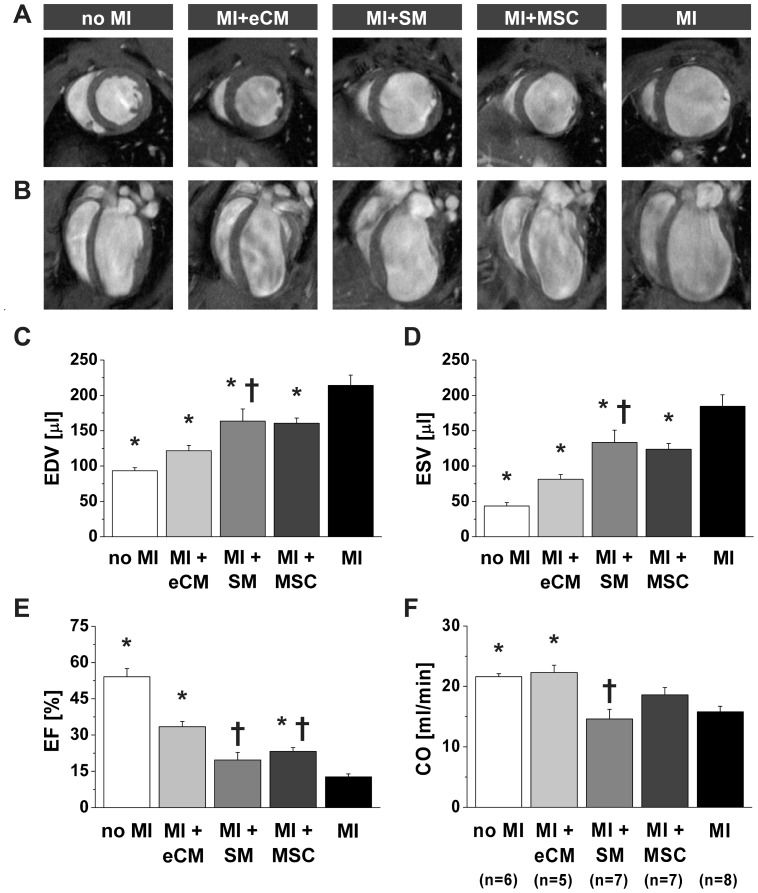
Quantification of cardiac function by MRI. Representative in vivo end-diastolic MR-images two weeks after myocardial infarction in A) short-axis (mid-LV) and B) long-axis orientation for each experimental group. Global cardiac morphology and function were obtained from in vivo cine MR-images: C) EDV, D) ESV, E) EF and F) CO. No MI: n = 6, MI+eCM: n = 5, MI+SM: n = 7, MI+MSC: n = 7 and MI: n = 8. *  =  p<0.05 vs. MI, †  =  p<0.05 vs. MI+eCM.

The effect of cell transplantation on various aspects of cardiac remodeling was evaluated two weeks after myocardial infarction with in vivo cine MRI. In Figs. 3A and B a collection of representative short- and long-axis end-diastolic cardiac MR-images is shown for the five groups. A decrease in LV dilation was observed by transplantation of either type of cells, which was in accordance with ex vivo histology (Fig. 1B).

Quantitative analysis of LV dimensions showed that both EDV (Fig. 3C) and ESV (Fig. 3D) were significantly decreased by transplantation of any of the three different cell types (p<0.05 vs. MI). Cell therapy caused a decrease in EDV from 214.1±14.6 µL (MI, n = 8) to 121.6±7.6 µL (MI+eCM, n = 5), 163.6±17.3 µL (MI+SM, n = 7) and 160.5±7.2 µL (MI+MSC, n = 7), whereas ESV decreased from 184.7±16.3 µL (MI) to 81.3±6.7 µL (MI+eCM), 133.3±17.4 µL (MI+SM) and 123.9±7.9 µL (MI+MSC). Importantly, the attenuation of LV dilation was most prominent when mice received eCMs (MI+eCM vs. MI+SM, p<0.05, and MI+eCM vs. MI+MSC, p<0.1).

An improvement of EF (Fig. 3E) after transplantation of all three cell types (MI+eCM 33.4±2.2%, MI+MSC 23.2±1.6%, MI+SM 19.7±3.1%) compared to MI mice (14.9±2.4%) could be demonstrated, which reached a significant level (p<0.05) for eCM and MSC groups. Within the cell transplantation groups again the increase of EF was significantly higher (p<0.05) after intramyocardial injection of eCMs compared to MSCs or SMs. The eCM therapy significantly restored CO (Fig. 3F) from 15.8±0.9 mL/min (MI) to 22.3±1.2 mL/min (MI+eCM). Importantly this value reached almost that of the wild type mice (21.6±0.5 mL/min). CO was not or only barely improved in mice that received SMs or MSCs (14.6±1.6 mL/min and 18.6±1.2 mL/min, respectively, p>0.05 vs. MI).

In summary, our morphological and functional analysis demonstrated that cell therapy with any of the three different cell types used could attenuate post-infarct ventricular dilatation. However, the positive effects were much more pronounced after intramyocardial eCM injection. Extensive functional MRI analysis also yielded impressive improvements in the eCM group and are therefore fully in line with the above reported histological findings.

### Regional myocardial contractility

**Figure 4:**
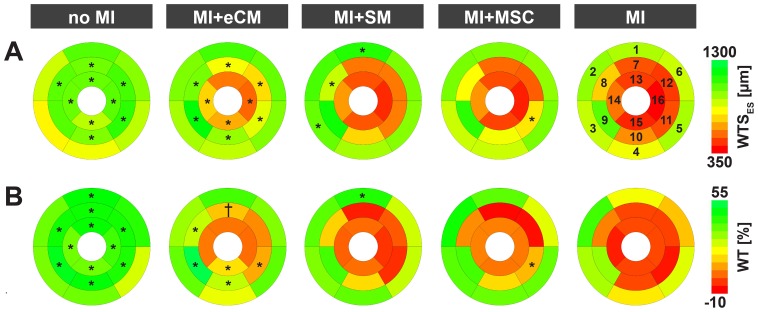
Assessment of regional contractile function by MRI. Regional contractile function two weeks after myocardial infarction, described by group-averaged A) end-systolic wall thickness and B) wall thickening after segmentation of the LV according to the 16 segment AHA model for mice with no MI (n = 6), MI+eCM (n = 5), MI+SM (n = 7), MI+MSC (n = 7) and MI (n = 8). *  =  p<0.05 vs. MI, †  =  p<0.1 vs. MI. The segment numbering is clarified in A).

In vivo MRI proved that eCMs, but not autologous stem cell treatment, significantly attenuates infarct size and significantly improves global function. To elucidate whether cellular cardiomyoplasty could also improve regional contractile function, changes in local LV wall thickness were studied throughout the cardiac cycle by exploiting the high contrast on in vivo cine MR-images between the myocardium and surrounding tissue. Figures 4A and B illustrate the mean regional end-systolic wall thickness (WTS_ES_) and wall thickening (WT) two weeks postoperatively in 16 segments of the standard AHA model. Both WTS_ES_ and WT were reduced by myocardial infarction at the apical and mid-LV level (p<0.05 no MI vs. MI).

Transplantation of eCMs resulted in a significant increase in the WTS_ES_ in all apical and mid-LV segments, except for the apical anterior segment (AHA segment #13) (Fig. 4A). In the apex, the mean WTS_ES_ of all segments (#13–16) increased from 447±33 µm (MI) to 628±54 µm (MI+eCM), whereas at the mid-LV level eCM treatment improved the mean WTS_ES_ of all segments (#7–12) from 650±92 µm (MI) to 970±94 µm (MI+eCM).

Moreover, eCM injection enhanced the WT at the apical as well as mid-LV level (Fig. 4B). In the apex, the mean WT (#13–16) increased from −3.8±2.3% (MI) to 4.6±3.9% by eCM transplantation (p<0.05). Furthermore, there was a significant (p<0.05) augmentation in the WT of the inferior segment (#15) from −3.1±2.1% (MI) to 13.1±4.7% (MI+eCM). At the middle of the left ventricle in the infarct border zone, WT was significantly enhanced by eCM transplantation in the anteroseptal (#8), inferoseptal (#9), inferior (#10) and inferolateral (#11) segments (p<0.05 vs. MI). Furthermore, in the anterior area (#7) a trend towards improved WT was found by eCM therapy (p = 0.07 vs. MI). The mean WT at the mid-LV level (#7–12) was increased from 2.5±5.1% (MI) to 20.3±8.0% (MI+eCM) (p<0.05). No significant improvements in WT were detected at the approximate site of eCM delivery (anterolateral segments #12, 13 and 16).

For SM and MSC transplantation no evident improvements in both WTS_ES_ and WT were observed. Therefore, eCMs had the unique potential to improve local myocardial contractile function, especially within the border zones at the mid-LV level.

### Long-term effects of cell engraftment to animal survival and cardiac function

**Figure 5:**
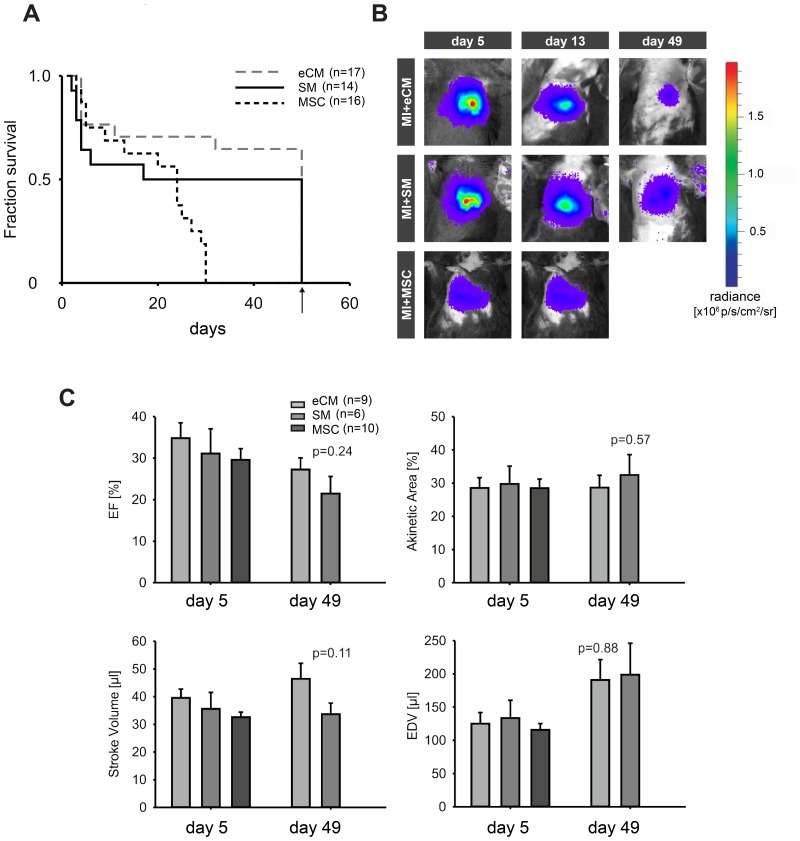
Long-term cell survival and treatment outcome. A) Survival curves of mice with myocardial infarction transplanted with eCMs (n = 17), SMs (n = 14) and MSCs (n = 16), followed 7 weeks postoperatively. B) In vivo longitudinal BLI of representative mice at 5, 13 and 49 days after transplantation of eCMs (top) SMs (middle) and MSCs (bottom) to monitor long-term cell survival in infarcted myocardium. C) Longitudinal evaluation of heart function with echocardiography at day 5 and 49 after transplantation of eCMs, SMs and MSCs.

The MRI analysis clearly proved a prominent functional benefit by the engraftment of eCMs. However, earlier studies suggested that such beneficial effects of cell replacement could be of transient nature [19]. For this, long-term effects of cell engraftment to animal survival and cardiac function were studied in a second series of mice up to 7 weeks postoperatively. We specifically compared eCMS to SMs and MSCs, because for the latter two cardiac function was significantly less augmented. In Fig. 5A, survival curves of treated mice clearly illustrate that long-term survival was dependent on the type of injected cells. Transplantation of eCMs significantly improved the survival of mice with myocardial infarction compared to MSCs (p<0.05). Seven weeks after cell transplantation, 65% (n = 11/17) of the mice that received eCMs completed the entire protocol, as opposed to 50% (n = 7/14) and 0% (n = 0/16) of the mice with SM and MSC transplantation, respectively. Especially, the low survival of MSC mice could be explained by the poor cell engraftment and the commonly observed high mortality in this permanent occlusion infarction model by extensive adverse remodeling [1], [20]. Most of the MSC mice showed clinical signs of heart failure, namely increasing shortness of breath due to pulmonary congestion prior to death. These observations are in line with our MRI data, were MSC mice showed the biggest infarcts.

Long-term cell engraftment was monitored by in vivo BLI of luciferase expression by viable transplanted cells. Signals originating from engrafted eCMs, SMs and MSCs at 5, 13 and 49 days after intramyocardial transplantation were detected (Fig. 5B). Over time, a slight signal decrease was observed for all three cell types, related to a loss of transplanted cells from the myocardium. In some hearts we have performed detailed quantitation of engrafted cells. Our analysis yielded for eCMs (n = 3) and SMs (n = 3) less than 5000 cells, thereby confirming their long-term survival, but also their steady decline in number and this is fully in accordance with the decrease in the bioluminescent signal (Fig. 5B).

The progression of cardiac remodeling after cell transplantation was followed with in vivo echocardiography up to seven weeks postoperatively. Figure 5C shows the EF, the akinetic area (as a measure for infarct size), stroke volume, and EDV at day 5 and 49. Functional parameters at day 5 were essentially the same between the groups, indicating that initial infarct size and cardiac function between groups were comparable. At day 49, heart function of mice treated with eCMs tended to be better than those treated with SMs (higher EF and larger stroke volume), although differences did not reach statistical significance; this may be also due to the fact that mice with SM and bad LV function had already died. These long-term echocardiographic data were in line with the MRI results two weeks postoperatively, since at this time point eCM-treated mice showed better LV function, which resulted in better survival numbers. Nevertheless, we could also observe a deterioration of LV function in eCM, this is consistent with earlier reports [21] and may be also related to a steady loss of grafted cells.

## Discussion

Transplantation of eCMs, SMs or MSCs in infarcted myocardium led to the following observations: 1) only eCM transplantation reduced the relative infarct size. 2) LV dilation was attenuated by any type of cell therapy, as reflected in decreased EDV and ESV, though most by eCMs. 3) eCM transplantation resulted in the largest improvement in global contractile function. 4) As hypothesized, local contractile function was only improved by eCMs. However, this effect was restricted to the infarct border zones. 5) Long-term follow-up showed a superior survival of eCM-treated mice compared to SMs and MSCs.

The ability of embryonic and autologous stem cells to improve the architecture of infarcted myocardium has been extensively explored [2]. However, somatic stem cells often fail to regenerate functional myocardium and contribute only through passive mechanisms to cardiac remodeling [22], [23]. Embryonic cardiomyocytes or stem cell derived cardiomyocytes that integrate electromechanically with native myocardium could provide the heart with new rhythmically contractile units [4], [19], [24], [25].

To study this topic, syngeneic GFP expressing murine embryonic cardiomyocytes (eCMs), or also clinically used somatic stem cells, namely SMs or bone-marrow-derived MSCs, were injected intramyocardially into infarcts. SMs and MSCs were not expected to differentiate into cardiomyocytes, but were able to form contractile myotubes or mesenchymal cells like fibroblasts, osteoblasts or lipocytes, respectively [2], [7], [8].

An in vivo multimodal imaging approach was used combining BLI to evaluate cell survival, and MRI and echocardiography to determine the effect of eCMs, SMs and MSCs on cardiac remodeling. Repetitive BLI of luciferase expression by the transplanted cells showed survival of eCMs and SMs up to seven weeks postoperatively (Fig. 5B). SMs were found to engraft better compared to the other cell types used and also to eCM (Fig. 5B). This is a consistent finding also in our earlier studies and probably can be related to the bigger size of the SMs. Dissociated eCMs are more or less round shaped and 20–30 µm in diameter. SMs are fusiform 20–30 µm in diameter and up to 200 µm long. Also SMs are better resistance to hypoxic conditions within the infarcted area. As shown before despite better initial engraftment of SM, eCM transplantation results, also short term, in better hemodynamic augmentation [4].

In time, a gradual loss of cells from the heart was observed with BLI, but functional benefits seen after two weeks were still detected seven weeks postoperatively [22], [23], [26], [27]. BLI has a high sensitivity to detect luciferase-expressing cells and was preferred over physical labeling of cells with MR-contrast agents such as iron oxides. Iron oxide labeling cannot report on cell viability and importantly several studies have observed MR hypoenhancement originating from leukocytes that phagocytized dead iron-laden cells [28]–[30]. Additionally, iron oxide disturbs the anterolateral myocardial contrast required for segmentation of the epi- and endocardium and interferes with LGE evaluation of infarct size [31]. MRI enabled detailed analysis of cardiac function and remodeling, because of its superior ability to visualize the endo- and epicardium compared to other imaging modalities [32]–[34].

Many studies have observed positive effects of cell therapies on global cardiac parameters [4], [11], [19], [22], [25], [26], [35]–[37]. It was hypothesized by Wall et al. that this could be attributed to a reduction in wall stress by the intramyocardial presence of compliant material [38]. Indeed, LV remodeling was significantly impaired by transplantation of all three cell types. For SMs and MSCs this was also observed, at least transiently, in clinical studies. However, this effect was most prominent in the eCM group (Figs. 3C–D), which indicates that the progression of cardiac remodeling also depended on cardiomyocyte-related cell features. This was supported by the finding that the volume fraction of chronically infarcted myocardium was only affected by eCM therapy (Figs. 2B–C). Importantly, this finding was not related to cell graft size, since approximately equal numbers were counted in eCM and SM transplanted hearts [39]. In addition, the endocardial areas of MR-signal hypointensities detected after eCM transplantation, which were absent after both SM and MSC therapy, do not correspond to regions of enhanced survival of native cells in the infarct as transmural infarcts were observed in all mice.

A plausible explanation for the smaller infarct size could be that eCMs prevented infarct expansion into the viable border zones. In the acute phase of myocardial infarction, infarct expansion is caused by cardiomyocyte slippage, which results in infarct wall thinning and dilation [40]. The alignment and electrical integration of eCMs with the native myocardium for synchronized contraction could limit sliding of cardiomyocyte bundles and might actively preserve local wall shear stress, which reduces systolic bulging and thus acute infarct expansion. In earlier studies, we and others have provided evidence that engrafted eCMs and human ES cell cardiomyocytes can couple electrically with the native myocardium, using genetic Ca^2+^ sensors combined with cell grafting experiments [4], [41]. This might prevent chronic progressive infarct extension into viable border zones, which is caused by stretch-induced myocyte apoptosis in areas that experience increased wall shear stress [42]. Thereby, transplantation of cells with a cardiomyocyte phenotype in infarcted myocardium might rescue the neighboring non-ischemic myocardium. This would also explain why eCM therapy showed most potential to maintain LV morphology and function (Figs. 3C–F).

Analysis of the local contribution of eCMs to the infarct and its border zones indeed showed that infarct wall thinning was less prominent after eCM therapy (Fig. 4A). However, the transplantation of contractile eCMs was not sufficient to restore wall thickening in the anterolateral infarct center (Fig. 4B). This is probably related to the relatively low survival of intramyocardial-injected eCMs in total and especially in the center of the lesion. Cells preferentially engrafted close to and within the less ischemic and still perfused infarct border zones. As shown in Fig. 4, WTS_ES_ and WT were significantly increased in these segments compared to non-treated hearts. To our opinion different mechanisms contribute to this phenomenon. Implanted eCMs act anti-apoptotic resulting in smaller infarct sizes, stimulate proliferation (proven by Ki67 stainings, data not shown), and augment the extracellular matrix as demonstrated for the extracellular matrix protein perlecan before resulting is less dilation [43]. After integration into the host tissue eCMs are able to couple electrically. Since engraftment predominantly happens within the border zone it is difficult to distinguish between active and passive effects. Nevertheless in the past force generation of transplanted murine eCMs within myocardial scar tissue was shown [3]. Whether primarily secretion of factors or proteins also of the co-transplanted cardiac fibroblasts, or also the ability to form cell-cell contacts to the host tissue plays a major role has to be cleared in future studies. Nevertheless, the combination of local effects on the lesion and global effects to the remaining LV myocardium resulted in significantly better hemodynamic performance of eCM treated hearts as depicted in Figs. 3E and F. These results clearly emphasize the necessity of a rhythmically contractile cell phenotype for cell replacement strategies in the infarcted as was previously suggested by Sakai et al. [11].

Long-term monitoring of cardiac function by echocardiography provided two major findings: Survival of the three populations was quite different (Fig. 5A). Most survivors were seen in the eCM group, while all MSC injected mice died during the first five weeks. Half of deaths after SM transplantation occurred during the first postoperative week. This is interesting as in clinical studies ventricular tachycardia (VT) were observed after SM transplantation and we could prove in the past increased malignant VT incidence during electrophysiological testing after SM transplantation in a murine infarct model [4], [44]. Furthermore, SM and MSC treated mice showed severely impaired LV geometry on MR-images. While survival of eCMs and SMs could still be demonstrated by BLI and histology seven weeks postoperatively (Fig. 5B), bone marrow derived cells were nearly absent four weeks postoperatively in previous studies [45]. With echo, clear differences of EF were seen in eCM and SM treated mice. Various other groups have also evaluated the long-term contribution of cell transplantation to cardiac function after myocardial infarction [19], [22], [23], [25]–[27], [30], [35]. Van der Bogt et al. reported improved contraction by SM therapy, but not by MSCs [22], [23]. Van Laake et al. observed non-persistent improvements in global function after transplantation of human embryonic stem cell-derived cardiomyocytes (ESC-CM) during a 12 week follow-up [19]. These conflicting results with the current study might be ascribed to differences in the origin of the cells used (adult vs. fetal, human vs. murine).

The importance of a cardiomyocyte cell phenotype for successful recovery of local contractile function is supported by a recent study by Qiao et al. [25]. They reported long-term improvements after two months in regional contractility of ischemia-reperfusion injured myocardium by ESC-CM therapy in rats. In analogy with our results, coupling of ESC-CMs by Connexin43 expression to native cardiomyocytes was observed and this electromechanical integration was probably responsible for improved infarct contractility.

The present study has some limitations. The mouse model of myocardial infarction by permanent ligation of the LAD has a given higher mortality than after e.g. cryolesion injury. This led, especially in the long-term experiments, to smaller group sizes. On the other hand, coronary ligation represents a more relevant physiological model of myocardial infarction. Also, individual anatomical variations of the coronary system in the mouse may result in significant variations in infarct size. We have therefore determined infarct size by echocardiography in all long-term experiments at 5 days after the infarction and injection of cells. These scans revealed no significant differences in the infarct size between the groups.

To conclude, this study provided convincing evidence that eCMs improved local and global contractile function after myocardial infarction. This drastically altered the course of global cardiac remodeling and prevented progressive infarct expansion into neighboring non-ischemic myocardium, which ultimately improved long-term survival. These effects were probably caused by the ability of eCMs to develop into contractile cardiomyocytes and form gap junctions and were therefore exclusively associated with eCM, but not SM or MSC therapy after myocardial infarction.
